# Non-invasive prenatal diagnostic test accuracy for fetal sex using cell-free DNA a review and meta-analysis

**DOI:** 10.1186/1756-0500-5-476

**Published:** 2012-09-01

**Authors:** Caroline F Wright, Yinghui Wei, Julian PT Higgins, Gurdeep S Sagoo

**Affiliations:** 1 PHG Foundation, 2 Worts Causeway, Cambridge, UK; 2 MRC Biostatistics Unit, Institute of Public Health, Robinson Way, Cambridge, UK

**Keywords:** Cell-free fetal DNA, Meta-analysis, Non-invasive prenatal diagnosis

## Abstract

**Background:**

Cell-free fetal DNA (cffDNA) can be detected in maternal blood during pregnancy, opening the possibility of early non-invasive prenatal diagnosis for a variety of genetic conditions. Since 1997, many studies have examined the accuracy of prenatal fetal sex determination using cffDNA, particularly for pregnancies at risk of an X-linked condition. Here we report a review and meta-analysis of the published literature to evaluate the use of cffDNA for prenatal determination (diagnosis) of fetal sex. We applied a sensitive search of multiple bibliographic databases including PubMed (MEDLINE), EMBASE, the Cochrane library and Web of Science.

**Results:**

Ninety studies, incorporating 9,965 pregnancies and 10,587 fetal sex results met our inclusion criteria. Overall mean sensitivity was 96.6% (95% credible interval 95.2% to 97.7%) and mean specificity was 98.9% (95% CI = 98.1% to 99.4%). These results vary very little with trimester or week of testing, indicating that the performance of the test is reliably high.

**Conclusions:**

Based on this review and meta-analysis we conclude that fetal sex can be determined with a high level of accuracy by analyzing cffDNA. Using cffDNA in prenatal diagnosis to replace or complement existing invasive methods can remove or reduce the risk of miscarriage. Future work should concentrate on the economic and ethical considerations of implementing an early non-invasive test for fetal sex.

## Background

Knowledge of the genetic status of the fetus in an on-going pregnancy gives couples the power to make an informed decision about their unborn child. When a fetus is known to have a particular genetic abnormality, a decision may be made either to choose termination or to continue with the pregnancy and take steps to provide appropriate care for the newborn child. Prenatal testing falls into two categories: screening and diagnosis. Prenatal screening is offered to all pregnant women as part of routine prenatal care to determine if the fetus is at substantial risk of having a particular disorder such as Down Syndrome or sickle cell anaemia. In cases deemed to be at high risk, prenatal diagnosis is offered to provide a definitive diagnosis and determine whether the fetus has inherited a disorder.

Prenatal genetic diagnosis is often used where there is a family history of a sex-linked disease. Most sex-linked diseases are recessive X-linked diseases caused by a particular mutation on the X chromosome. The disease is normally manifested only in males, who carry a single X chromosome, whilst in females the normal allele on the second X chromosome compensates for the diseased allele. The most common X-linked recessive diseases include haemophilia (a blood clotting disorder) and Duchenne muscular dystrophy (a progressive muscle wasting disease), although numerous others can result in severe conditions. Whilst each disease is individually relatively rare, it has been estimated that in combination they occur in around 5 in 10,000 live births [1].

In the UK, sex-linked diseases are usually diagnosed through referral to a clinical geneticist when there is a known family history of a particular disease. Although fetal sex can often be determined using an ultrasound scan of the fetus in the second or third trimester, a definitive prenatal diagnosis can only be made through invasive testing in which a sample of fetal cells is physically removed from the uterus for genetic analysis, using either chorionic villus sampling (CVS) at 11–14 weeks gestation, or amniocentesis from 15 weeks gestation. Both these invasive techniques carry a small but significant risk of miscarriage (1-2%) [2] and although currently the gold standard for prenatal diagnosis, many women are reluctant to undergo invasive testing. However, there are substantial advantages to earlier diagnosis. Where future management might involve a decision to terminate the pregnancy, early termination carries fewer risks (being medically induced or involving surgical vacuum aspiration), whilst late termination (at more than 14 weeks) may require the induction of labour, potentially causing significantly greater physical, emotional and psychological complications.

Due to the risk of miscarriage with these traditional prenatal diagnostic methods, enormous interest has arisen in the field of non-invasive prenatal diagnosis (NIPD). In 1997, Lo *et al.*[3] demonstrated the presence of fetal DNA in the maternal blood, opening the possibility that a simple blood test could provide a non-invasive method for prenatal diagnosis. Fragments of cell-free fetal DNA (cffDNA) originating from the placenta are detectable in the maternal blood stream [3] from 5 weeks gestation until birth [4], when they are rapidly cleared from the circulation and are undetectable within 2 hours [5]. It has been proposed that cffDNA could be used for non-invasive prenatal diagnosis. However, cffDNA only comprises around 3% to 6% [4], although up to 10% has also been reported [6], of the total cell-free DNA in the maternal circulation during pregnancy, the rest being maternal in origin. Therefore, distinguishing, or ideally isolating, fetally derived cell-free DNA in an overwhelming background of maternal DNA is a significant technical challenge due to the high level of molecular similarity between it and the maternally derived cell-free DNA. As a result, a number of different protocols have been developed to extract the cell-free DNA from a blood sample and analyze it for fetal specific sequences, usually with real time quantitative polymerase chain reaction (qPCR).

To date, the most advanced application of cffDNA for prenatal diagnosis is fetal sex determination for pregnancies at high risk of an X-linked disease (or certain masculinising endocrine disorders), in order to reduce the need for invasive testing. This is achieved through selective amplification and detection of Y chromosome sequences not otherwise present in the mother, most commonly the sex determining region Y (SRY), but sometimes using the testis specific protein Y linked 1 (also known as DYS14). The fetus is presumed to be female if no Y chromosome DNA can be detected. This technology is already being translated into a clinical setting and is used routinely in some clinics in the UK and elsewhere, and has been shown to reduce the need for invasive testing by 45% [7]. In addition, several companies currently offer commercial mail-order fetal sexing using cffDNA from a home finger prick sample.

Since 1997, a number of large studies examining the accuracy of prenatal fetal sex determination using cffDNA have been published [8,9] as well as many smaller ones (reviewed by Avent & Chitty [10]). Devaney *et al.*[11] have recently published a systematic review and meta-analysis documenting the overall test performance of non-invasive fetal sex determination using cffDNA including data from 57 studies and approximately 6,500 pregnancies. This review was limited however to English language publications and only searched for journals listed in PubMed. Here we report our review and meta-analysis of the wider literature in order to further evaluate the use of cffDNA in the maternal circulation for non-invasive prenatal determination of fetal sex.

## Methods

### Eligibility of studies

We sought all studies in which Y chromosome cell-free fetal DNA (not fetal cells) was extracted from a maternal blood sample and used for sex determination. Pregnant women participating in the studies had to be greater than 5 weeks gestation [4]. The gold standard against which non-invasive prenatal diagnosis of fetal sex is measured is the baby’s sex on examination at birth, although we also included studies in which it was determined during pregnancy by amniocentesis or CVS. We considered only studies in which data were presented that allowed a cross-tabulation of sex determination for cffDNA against the reference standard, permitting estimation of sensitivity and specificity.

### Search strategy

We applied a sensitive search of multiple bibliographic databases in March 2010 using text words and MeSH terms, adapting them for each different database. The databases searched and the search terms used in PubMed are listed in the supplementary information. The search was not limited to English language publications or publication type. A filter for diagnostic studies was not applied. Investigators in the field were also contacted for any data not explicitly included in the publications. No reference was made to gender or sex in our search strategy, as many studies that focus on using cffDNA for alternative diagnoses (e.g. RhD, aneuploidy, inherited single-gene disorders, pre-eclampsia, etc.) also test for fetal sex as part of their protocol. However, we excluded the small number of studies using massively parallel sequencing for Down Syndrome [12,13] or fetal profiling [6] and looked only at targeted tests.

We attempted to identify cumulative papers which reported data from the same dataset, and contacted authors to obtain clarification of the overlap between data presented in these papers, in order to prevent test data from the same women being analyzed more than once.

### Study selection

**Figure 1:**
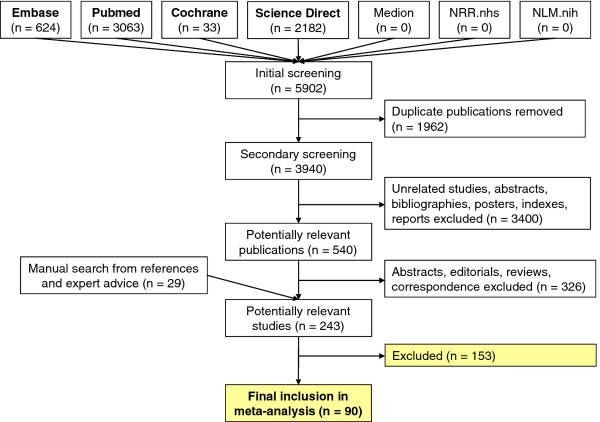
Flow chart of studies identified by the search strategy and the process of eligibility and inclusion.

All relevant articles identified by the search were scanned on the basis of title, keywords and abstract (where available). Articles were rejected on the initial screen if the reviewer (CW) could determine that the article clearly did not match the eligibility criteria or if the study was published before cffDNA was discovered (1997) [3]. Where a title or abstract could not be rejected with certainty, the full text of the article was obtained for evaluation. The full texts of all relevant articles identified by reference searching were also obtained. Two reviewers (CW and GS) then independently assessed the eligibility of studies for inclusion in the review. If disagreements were not resolved by discussion, a third reviewer (JH) was consulted. Figure 1 presents the number of articles identified by our search strategy along with the process of selecting studies into our review.

### Data collection

We followed methods suggested by the Cochrane Screening and Diagnostic Tests Methods Group [14]. The PRISMA checklist is attached as Additional file 1. Data were extracted independently by two reviewers, one onto a pre-piloted paper data extraction form (GS) and one onto an equivalent computer database in Microsoft Excel (CW). Differences between extracted data were resolved by discussion and, if necessary, through consultation with a third reviewer (JH). All reviewers participating in the study participated in the pilot of the data extraction form with 10 randomly selected articles.

We extracted descriptive data on primary author, year of study, journal of publication, number of participants including information on gestation, methods used for DNA isolation and preparation where available including blood sample volume, cffDNA sequence detected and the detection method used. We examined each included study for potential major threats to validity. Attempts were then made to obtain any missing or unclear data by contacting the authors of each article.

For each study, we extracted a 2 × 2 contingency table in which all individuals were classified as falling into one of the following categories: true positive (TP), false positive (FP), true negative (TN) or false negative (FN), according to the test results. We took a true positive to mean the test-detected presence of Y chromosomal material in a confirmed male baby; conversely, a true negative was the non-detection of any Y chromosomal material (plus the detection of control material) in a confirmed female baby. Where available, we also extracted the number of tests with inconclusive or uncertain result, though this information was not used in the formal meta-analysis.

Some of the test results were recorded with specified trimester (1, 2 or 3) and a few of the data were recorded every prenatal week from the 5^th^ to 13^th^ week. The remaining (majority) test results were recorded without specifying in which trimester or week the test was undertaken. The primary dataset contained one 2 × 2 table from each study.

### Data analysis

We performed meta-analysis to estimate summary sensitivity and specificity using a bivariate model [15]. This jointly analyses each sensitivity and specificity pair, incorporating the anticipated negative correlation between them through a random-effects approach [15]. The model has two levels. At the first level, the numbers of true positives and true negatives are assumed to follow independent binomial distributions with parameters representing sensitivity and specificity, respectively; at the second level, the logit transformations of sensitivity and specificity are assumed to follow a bivariate normal distribution across studies, which allows for heterogeneity and for the correlation between sensitivity and specificity. We derived prediction intervals from the results of the analysis [16]. Forest plots of sensitivity and specificity used an asymptotic standard error and an assumption of normality on the logit scale. We used these approximate results to assess the contribution of each study to the total heterogeneity for sensitivity and specificity separately, as described by Thompson [17], in order to inform sensitivity analyses.

We performed further bivariate meta-analyses to investigate whether the accuracy of cffDNA testing changes over time during a pregnancy. A second dataset was constructed to examine the possible effect of trimester on test accuracy. Only studies providing specific information on trimester of test were included in this dataset. We analyzed data from each trimester separately, and compared formally them using a meta-regression analysis with a categorical covariate representing trimester. A third dataset was constructed to examine the possible effect of week on test accuracy. Only studies providing specific information on the week of the test within the first trimester were included in this dataset; 2 × 2 tables in which there were no true males or no true females were omitted. We again used bivariate meta-regression, with a linear effect of week on the logit scale. We used posterior summary statistics for coefficients in the regression to illustrate fitted changes in sensitivity and specificity from five to 13 weeks.

#### Investigation of heterogeneity

To investigate the variation in diagnostic accuracy across the studies, we performed meta-regression by incorporating covariates into the bivariate model. In order to assess different techniques used for testing, we used as covariates blood sample (recorded as plasma or serum), sequence detected (SRY or DYS14 or other), detection technique (qPCR or other), extracted blood volume, publication year. Covariates were incorporated into the model through the second level, such that the logit sensitivity and logit specificity were separately regressed on the available covariates. We chose plasma, SRY, and qPCR as baselines for the categorical covariates; the coefficients for these covariates are therefore the comparison of other categories with the baseline. We performed the analyses both with all covariates included simultaneously in the model, and with each covariate included one at a time. Although there were very few instances of multiple versions of the test being used within a study, if women were tested on more than one covariate, we selected data from plasma in preference to serum, and SRY in preference to DYS14 as they were the most commonly reported. We did not evaluate these differences, due to a combination of lack of power and lack of cross-tabulated data.

There were some missing data in both categorical and continuous covariates. We employed multiple imputation techniques to impute the missing, making a ‘missing at random’ assumption. A missing value for a categorical variable was imputed from a categorical distribution with parameter p=1/c, where *c* is the total number of different categories of the variable. A missing value for a continuous variable was imputed from a normal distribution with mean and standard deviation equal to those observed for that variable from non-missing values.

#### Implementation

The estimates of sensitivity and specificity from the bivariate model as well as the coefficients for the covariates were computed in a Bayesian framework using Markov chain Monte Carlo simulation with publicly available software (WinBUGS) [18]. We added 0.1 to empty cells in the 2 × 2 tables. Prior distributions were selected so as to be vague in order to emulate a frequentist analysis: normal with mean 0 and precision 0.00001 for means and coefficients, and wishart(R, df), with R = identify matrix and df = 2 for between-study covariance matrices. For the main analyses we report results based on 10,000 draws of the Markov chain, of which the first 10% were discarded as burn-in. For meta-regression analyses we used 50,000 draws, of which the first 10% were discarded. In all analyses, trace plots for the Markov chain show good mixing of the chains, confirming the convergence of Markov chains to their posterior distributions. Credible intervals from these analyses may be interpreted approximately as confidence intervals. Forest plots were drawn using Review Manager [19].

## Results

### Study characteristics

Ninety studies [3,4,7-9,13,20-103], incorporating 9,965 pregnancies, were identified that met the eligibility criteria ( Additional file 2: Table S1). Fifty studies were located in Europe [3,7,8,20,25-27,29-32,35,37-40,42,46-48,50,52,53,57,59-81,94,98,103], 26 from Asia [4,9,23,24,28,34,36,44,54-56,58,82-86,88,89,91-93,96,100-102], eight from North America [13,21,22,43,51,87,97,99], two from multiple locations [49,90] and a further four from around the rest of the world [33,41,45,95]. Gestational ranges for the pregnant women varied across the studies, as did the amount of blood taken and the volume actually used for extracting DNA (see Additional file 2: Table S1). The vast majority of studies extracted DNA from blood plasma (n = 74), with nine studies using blood serum [20,23,35,50,59,64,77,78,97], five studies using both plasma and serum [3,4,65,95,103], and two studies not stating which was used [7,62]. Real-time quantitative (q) PCR was the most commonly applied detection technique (n = 61), with nested PCR used in 15 studies [25,28,34,45,56,58,62,72,76,85,93,96,100,102,103], standard PCR used in a further eleven studies [3,20,23,24,29,31,65,84,88,95,101], and other methods used in three studies [47,55,82]. SRY alone was used for fetal sex determination in 49 studies, DYS14 alone in 17 studies [3,9,13,23,28,33,41,42,46,49,57,58,65,68,76,100], both SRY and DYS14 in a further five studies [7,27,32,40,69], with 19 other studies [20,21,24,31,43,51,62,63,72,77,82,84,87,88,91,94,97,101,102] using different markers including amelogenin (n = 6) [31,62,63,72,91,102] or a combination of markers. Only four studies specifically recorded inconclusive results or failed tests [7,27,29,52]. We excluded from the meta-analysis data from studies that had no record on foetus sex.

In total we had available 115 2 × 2 contingency tables from the 90 independent studies, containing 10,587 fetal sex test results. In order to create our primary data set of one table per study, we made the following decisions. First, where trimester information was available for a study but when different women were tested in different trimesters, we summed cell counts across trimesters to obtain the total (82 studies). In one study, results for the same women were reported for each trimester separately. For this study we selected data from the first trimester only. In seven studies, more test results were reported than there were women (i.e. at least some women contributed multiple tests) and we were unable to obtain woman-specific test results. For these studies, we included all test results in the analysis, assuming independence of test results within the study. We acknowledge that this may have resulted in some spurious precision, but the proportion of information in the meta-analysis to which this applies is small.

The second dataset (investigating the effect of trimester) comprises 52 2 × 2 tables of test results from 35 studies incorporating 4,467 fetal sex test results where the trimester of testing was specified: 26 studies contributed tables from the first trimester, 15 from the second and nine from the third. The third dataset (investigating the effect of week) comprises 55 2 × 2 tables from 13 studies covering 1,001 fetal sex test results where the week of testing during the first trimester was specified.

### Sensitivity and specificity

**Figure 2:**
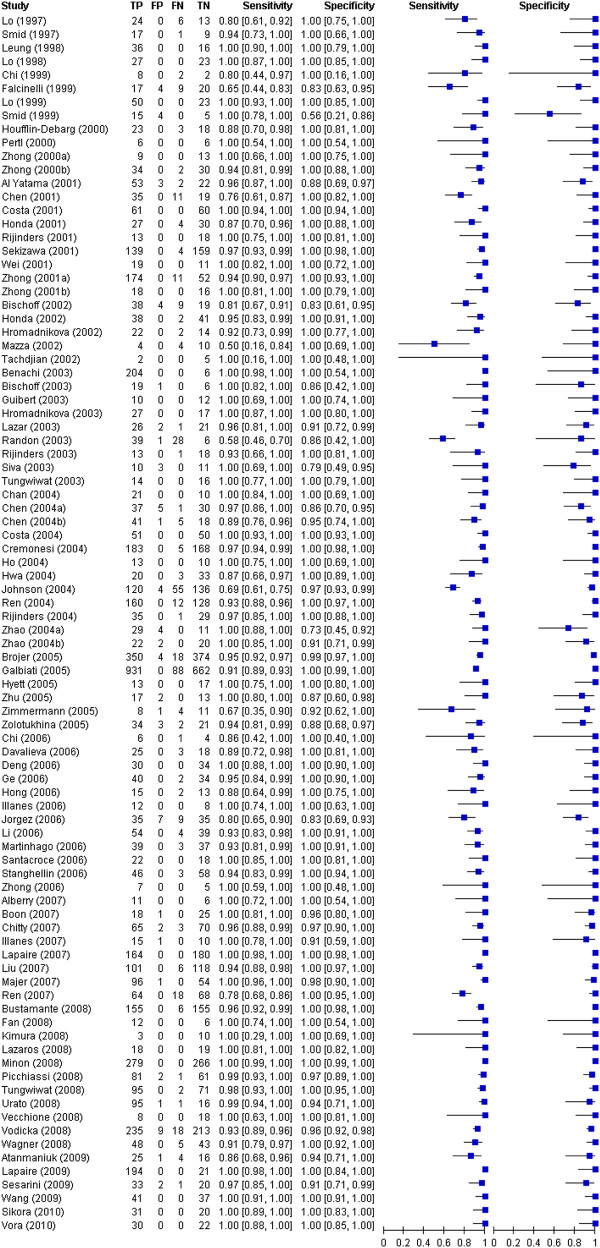
Individual study estimates of sensitivity and 1-specificity of cffDNA diagnosis of fetus sex, ordered by year of publication.

**Figure 3:**
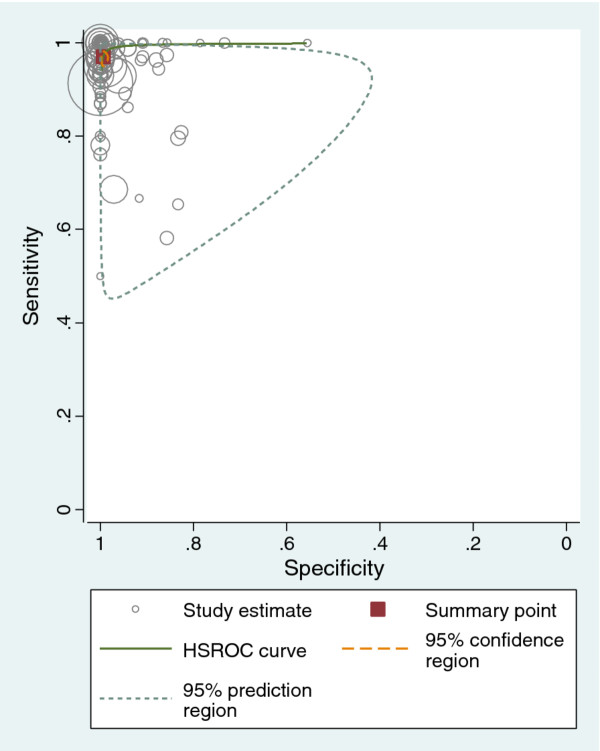
**ROC plot of sensitivity against (1 − specificity) for 90 studies of the diagnosis of fetal sex by cffDNA. Each point on the graph represents a pair of sensitivity and specificity from a study.** The more weight the study is given, the larger the point. Summary sensitivity and specificity is marked by a red square.

Study-level estimates of sensitivity and specificity for all studies are presented in Figure 2. Figure 3 gives a graphical display of these results, with sensitivity (true-positive rate) on the vertical axis and the 1 − specificity (false-positive rate) on the horizontal axis. Most of the points cluster around the top left of the graph, indicating the high accuracy of the test. The prediction interval illustrates the upper and lower limits of where any future pair of sensitivity and specificity is expected to lie.

In the primary bivariate meta-analysis, involving 90 studies with 10,587 tests, average sensitivity was 96.6% (95% CI from 95.2% to 97.7%) and average specificity was 98.9% (95% CI from 98.1% to 99.4%). Table 1 shows the summary estimates from these bivariate meta-analyses, along with corresponding results from separate analyses for each trimester when this information was available. Average sensitivity and specificity did not vary markedly by trimester (see Table 1). Test accuracy was marginally higher in the second trimester, but no statistically significant differences were found in the meta-regression.

**Table 1:** Summary sensitivity and specificity based on bivariate model with no covariates

**Data**		**Median Sensitivity (95% CI)[95% PI]**	**Median specificity (95% CI)[95% PI]**	**Heterogeneity SD on logit scale (sens)**	**Heterogeneity SD on logit scale (spec)**
Primary analysis (n = 10,587, 90 studies)		96.6 (95.2, 97.7) [64.4, 99.8]	98.9 (98.1, 99.4) [75.8, 100.0)	1.4	1.7
Breakdown by trimester (n = 4,467, 35 studies)	First trimester (n = 2,244, 26 studies)	95.0 (92.2, 97.3) [71.7, 99.4]	98.8 (97.0, 99.7) [64.0, 100.0)	1.0	1.7
	Second trimester (n = 1,662, 15 studies)	98.2 (95.2, 99.6) [64.9, 100.0]	99.5 (98.2, 100.0) [77.0,100]	1.6	1.6
	Third trimester (n = 561, 9 studies)	96.6 (86.6, 99.7) [28.0, 100.0]	99.0 (95.4, 99.9) [70.6, 100.0]	1.8	1.3

CI = credible interval.

PI = prediction interval.

SD = standard deviation.

Two studies contributed substantially to heterogeneity in sensitivities across studies [22,26]. After excluding these studies from the bivariate analysis, overall sensitivity and specificity were very similar. A more marked effect was seen for the third trimester on omission of these studies, in which sensitivity increased from 96.6% to 97.8% (95% CI from 92.2% to 99.7%; prediction interval from 65.4% to 100.0%), and specificity from 99.0% to 99.4% (95% CI from 96.4% to 100.0%; prediction interval from 87.7% to 100.0%).

A small, but not statistically significant, improvement in diagnostic test accuracy can be seen over prenatal week (from 5th to 13th) in Additional file2: Table S2, based on the limited data available. At week 5, the sensitivity and specificity are 93% (95% CI from 84% to 98%) and 95% (95% CI from 87% to 99%) respectively; at week 13, the sensitivity and specificity are 98% (95% CI from 95% to 99%) and 99% (95%CI from 96% to 100%) respectively. The odds ratio for sensitivity and specificity are 1.19 and 1.21 per additional week respectively.

Of the four studies that reported inconclusive results or failed tests, failures represented 11-24% of tests, these pregnancies were generally re-tested at a later gestational age using a second sample [7,27,29,52]. There were several reported reasons for inconclusive, false negative and false positive results including blood samples not being processed appropriately within 48 hours of collection, poor quality blood serum or plasma, variable or low concentrations of cffDNA within the maternal blood samples collected and tested, and the diagnostic threshold used to determine fetal sex.

### Investigation of covariates

Summary statistics for the analyses of covariates and results of the meta-regression analyses investigating other covariates in the primary data set (one 2 × 2 table per study) are shown in Additional file 2: Table S3. The use of serum for DNA extraction rather than plasma may increase the accuracy of the test; in unadjusted models, the odds ratios for serum versus plasma are 4.8 (95%CI 1.2 to 20.7) and 6.2 (95%CI 0.84 to 77) for sensitivity and specificity, respectively. These correspond to improvements in sensitivity from 96% to 99%, and in specificity from 99% to virtually 100%. There was no discernable difference between using SRY or DYS14 as the DNA marker for fetal sex. The use of qPCR improved specificity compared with other lab-based detection techniques (though note that no studies using next generation sequencing platforms were included). The other lab techniques (including conventional PCR and nested PCR) decrease the sensitivity and specificity to 95% and 96%, respectively. Increasing the volume of blood that DNA is extracted from may increase both the specificity and sensitivity. Finally, there is an indication of an improvement in sensitivity over time.

## Discussion

This review and meta-analysis of non-invasive prenatal determination of fetal sex using cffDNA in maternal blood, incorporates 10,587 tests and demonstrates the test to be highly accurate in terms of both sensitivity and specificity. The overall average sensitivity of using cffDNA to determine fetal sex is 96.6% and the overall specificity is 98.9%. These vary very little with trimester or week of testing, indicating that the performance of the test is reliably high. The most commonly used method for detection and identification of cffDNA specific to the Y chromosome was qPCR, which demonstrated increased specificity when compared with other methods. A number of Y chromosome markers are available for detection purposes. Our analyses did not show a discernable difference between SRY and DYS14, but did suggest that these lead to slightly better specificity than the other markers that had been used in the studies. The majority of studies also used fetal DNA extracted from plasma, and there is some evidence that using fetal DNA extracted from serum may produce slightly higher sensitivity and specificity. However, although these findings produced statistically significant 95% credible intervals, the confidence intervals were very wide and so we would caution against over-interpretation of the findings. Previous work has shown that similar levels of cffDNA are detected in both plasma and serum [4] and laboratories often use automated and optimized methods for DNA extraction regardless of whether they use serum or plasma. Laboratories are also likely to try both the DYS and SRY markers and settle with the marker that gives them the most reliable and reproducible result in their setting.

When compared to the systematic review and meta-analysis published recently by Devaney *et al.*[11] the current review utilized data on an additional 33 studies and ~3,500 pregnancies (90 studies v 57 studies and 9,965 pregnancies v 6,541 pregnancies). Both reviews show test performance to be reliably high with the increase in data presented in the current review showing small increases in both sensitivity (96.6% compared with Devaney’s 95.4%) and specificity (98.9% compared with Devaney’s 98.6%).

One limitation of this study was our inability to properly evaluate the proportion of inconclusive or uncertain results, which we know to be problematic with this technique and may vary with gestational age [7,104]. However, in the case of an inconclusive test result performed early in pregnancy, it would still be possible to retest at a later date. In addition, all literature-based reviews are at risk of publication bias due to the suppression of unwanted findings. We searched extensively for studies and contacted experts in the area, but cannot rule out the possibility that our sample of studies is not fully representative.

The main implications for this test in relation to the current clinical pathway would be that invasive testing can be avoided in the case of a diagnosed female fetus. If a female were to be incorrectly classified as male (false positive), there would be no change to the current clinical pathway. If a male fetus is incorrectly classified as a female (false negative), or if a fetus is unclassified after the first test, then invasive testing would be delayed potentially resulting in reduced quality of care.

Genetic testing of DNA extracted by amniocentesis or chorionic villus sampling is currently routine for prenatal testing. Both of these procedures take place later in pregnancy than would be required by a methods using cffDNA, and both carry a small but significant risk of miscarriage, something that cffDNA does not. The results of both this review and meta-analysis and the analysis by Devaney *et al.*[11] show that the diagnostic test of fetal sex determination using cffDNA is expected to be close to 100%, it can be done early in pregnancy, and that the test itself carries no risk of miscarriage suggesting that we should seriously consider adopting this test as the new gold standard first-line test for pregnancies at risk of an X-linked condition. The test has been successfully used from 7 weeks of gestation in some NHS laboratories in the UK since 2003 following audit results [104,105], where it has already reduced the need for invasive diagnostic testing in high risk pregnancies.

## Conclusions

Based on the updated data provided in this review and meta-analysis we conclude that fetal sex can be determined with a high level of accuracy by analyzing cffDNA after 5 weeks of gestation. It is hoped that use of this method for non-invasive prenatal diagnosis could also be extended for single gene disorders and although testing is being developed, it is not currently offered on any routine basis within Europe. Advances in genomic methodology and technology continue to make tremendous progress and these advances need faster and timelier translation into clinical practice in order to provide couples with greater reproductive choice. Further research is needed into the logistical requirements, the economic considerations (including a possible value of information analysis) and the ethical implications of offering an early non-invasive test for fetal sex [106].

## Competing interests

There are no competing interests.

## Authors’ contributions

CFW: design of study, collection and interpretation of data, writing of manuscript. YW: analysis and interpretation of data, writing of manuscript. JPTH: design of study, analysis and interpretation of data, writing of manuscript. GSS: design of study, collection and interpretation of data, writing and finalization of manuscript. All authors are in agreement with the final version of the manuscript.

## Supplementary Material

Additional file 1 Prisma 2009 Checklist. Click here for file

Additional file 2 **Search terms as used in PubMed to search MEDLINE. 2). Table S1:** Studies identified in this review and their main characteristics, ordered by year of publication. 3) **Table S2:** Summary statistics from posterior distributions from bivariate meta-regression analysis investigating the effect of week of test (standardized). 4) **Table S3:** Summary statistics from posterior distributions from bivariate meta-regression analyses using all studies (n = number of 2 × 2 tables constructed from included studies). Click here for file
