# Supplementary material for: Non-invasive prenatal diagnostic test accuracy for fetal sex using cell-free DNA a review and meta-analysis
Source: BMC Res Notes. 2012 Sep 1;5:476. doi: 10.1186/1756-0500-5-476 (PMC3444439; doi:10.1186/1756-0500-5-476)
Supplement: Additional file 2 — Search terms as used in PubMed to search MEDLINE. 2). Table S1: Studies identified in this review and their main characteristics, ordered by year of publication. 3) Table S2: Summary statistics from posterior distributions from bivariate meta-regression analysis investigating the effect of week of test (standardized). 4) Table S3: Summary statistics from posterior distributions from bivariate meta-regression analyses using all studies (n = number of 2 × 2 tables constructed from included studies). [file 1756-0500-5-476-S2.docx]

**Supplementary Information**

***Search strategy***

Databases searched:

- MEDLINE via PubMed
- EMBASE via Dialog Datastar
- The Cochrane Library
- MEDION (http://www.mediondatabase.nl/)
- The National Research Register (http://www.nrr.nhs.uk/)
- Health Services Research Projects in Progress Database (http://www.nlm.nih.gov/hsrproj/)

Text words and MeSH terms used for PubMed Search:

free fetal DNA OR cell-free fetal DNA OR cell free fetal DNA OR free fetal nucleic acid* OR cell-free fetal nucleic acid* OR free fDNA OR cell-free fDNA OR cell free fDNA OR cffDNA OR ccffDNA OR ffNA OR cffNA OR non-invasive prenatal diagnosis OR noninvasive prenatal diagnosis OR non invasive prenatal diagnosis OR NIPD

Supplementary Table 1: Studies identified in this review and their main characteristics, ordered by year of publication.

| **Author (Year)** | **Country** | **Number of pregnancies** | **Number of tests** | **Gestation range** | **DNA extraction methodology** | | | **DNA detection methodology** | |
| --- | --- | --- | --- | --- | --- | --- | --- | --- | --- |
|  |  |  |  |  | **Extracted blood volume** | **Volume used for DNA extraction** | **Blood component used for DNA extraction** | **DNA detection technique** | **Sequence detected** |
| Lo (1997) [3] | UK | 43 | 43 | 12 – 40 | 5 - 10 | 0.2 | Plasma and serum | PCR | DYS14 |
| Smid (1997) [76] | Italy | 27 | 27 | 6 – 36 | 10-15 | NS | Plasma | Nested PCR | DYS14 |
| Leung (1998) [36] | Hong Kong | 52 | 52 | Up to 34 | 10 | 0.4-0.8 | Plasma | qPCR | SRY |
| Lo (1998) [4] | Hong Kong | NS | 50 | 11 – 43 | 10-20 | 0.4-0.8 | Plasma and serum | qPCR | SRY |
| Chi (1999) [58] | China | 12 | 12 | 12 – 40 | NS | NS | Plasma | Nested PCR | DYS14 |
| Falcinelli (1999) [62] | Italy | 50 | 50 | 7 – 11 | 10-15 | NS | NS | Nested PCR | Amelogenin |
| Lo (1999) [90] | US and China | 73 | 73 | 12 – 21 | 5-24 | 0.4-0.8 | Plasma | qPCR | SRY |
| Smid (1999) [29] | Italy | 27 | 24 | 7 – 32 | 6 | NS | Plasma | PCR | DYS14 |
| Houfflin-Debarge (2000) [65] | UK | 65 | 44 | 10 – 41 | 20 | 0.2 | Plasma and serum | PCR | DYS14 |
| Pertl (2000) [91] | Japan | 12 | 12 | 37 onwards | 5-10 | 1 | Plasma | qPCR | Amelogenin |
| Zhong (2000a) [25] | Switzerland | 22 | 22 | 10 – 21 | 15 | 0.4 | Plasma | Nested PCR | SRY |
| Zhong (2000b) [79] | Switzerland | 16 | 66 | 9 – 42 | 5 | 0.4 | Plasma | qPCR | SRY |
| Al Yatama (2001) [45] | Kuwait | 80 | 80 | 7 – 40 | 5-10 | 0.2 | Plasma | Nested PCR | SRY |
| Chen (2001) [55] | China | 65 | 65 | NS | 5 | NS | Plasma | Probe microplate hybridization | SRY |
| Costa (2001) [59] | France | 121 | 121 | 8 – 14 | 5 | 0.4 | Serum | qPCR | SRY |
| Honda (2001) [84] | Japan | 61 | 61 | 10 – 17 | 4 | 0.8 | Plasma | PCR | DYS14 and DYZ3 |
| Rijinders (2001) [75] | The Netherlands | 45 | 45 | 8 – 17 | 10 | 2 | Plasma | qPCR | SRY |
| Sekizawa (2001) [9] | Japan | 302 | 302 | 7 – 16 | 10 | 1.5 | Plasma | qPCR | DYS14 |
| Wei (2001) [99] | USA | 30 | 30 | 9 – 29 | NS | 5 | Plasma | qPCR | SRY |
| Zhong (2001a) [48] | Switzerland | 237 | 237 | 11 – 34 | NS | NS | Plasma | qPCR | SRY |
| Zhong (2001b) [80] | Switzerland | 34 | 34 | 13 – 17 | 15 | 0.4 | Plasma | qPCR | SRY |
| Bischoff (2002) [21] | USA | 70 | 70 | 10 – 23 | NS | NS | Plasma | qPCR | DYS1 |
| Honda (2002) [23] | Japan | 81 | 81 | 5 – 10 | 6-9 | 2 | Serum | PCR | DYS14 |
| Hromadnikova (2002) [30] | Czech Republic | 37 | 38 | 15 – 22 | 5 | 0.4 | Plasma | qPCR | SRY |
| Mazza (2002) [72] | Italy | 18 | 18 | 7 – 12 | 10-20 | NS | Plasma | Nested PCR | Amelogenin |
| Tachdjian (2002) [78] | France | 7 | 7 | 9 – 12 | 5 | 0.4 | Serum | qPCR | SRY |
| Benachi (2003) [50] | France | 363 | 363 | NS | 5 | 0.4 | Serum | qPCR | SRY |
| Bischoff (2003) [51] | USA | 26 | 26 | NS | NS | 0.112 | Plasma | qPCR | DYS1 |
| Guibert (2003) [64] | France | 22 | 22 | 7 – 13 | 5 | 1 | Serum | qPCR | SRY |
| Hromadnikova (2003) [66] | Czech Republic | 44 | 44 | 10 – 18 | 5 | 0.4 | Plasma | qPCR | SRY |
| Lazar (2003) [70] | Hungary | 50 | 50 | 11 – 22 | 6 | 0.8 | Plasma | qPCR | SRY |
| Randon (2003) [26] | Norway | 22 | 74 | 13 – 38 | 5 | 0.8 | Plasma | qPCR | SRY |
| Rijinders (2003) [73] | The Netherlands | 31 | 31 | 10 – 18 | 10 | 1-2 | Plasma | qPCR | SRY |
| Siva (2003) [95] | Australia | 24 | 24 | 11 – 18 | 9 | 0.4 | Plasma and serum | PCR | SRY |
| Tungwiwat (2003) [96] | Thailand | 30 | 30 | 7 – 32 | 2 | 0.2 | Plasma | Nested PCR | SRY |
| Chan (2004) [54] | Hong Kong | 31 | 31 | NS | 10-15 | 2 | Plasma | qPCR | SRY |
| Chen (2004a) [34] | China | 73 | 73 | 26 – 41 | NS | NS | Plasma | Nested PCR | SRY |
| Chen (2004b) [56] | China | 65 | 65 | 13 – 27 | 5 | 0.2 | Plasma | Nested PCR | SRY |
| Costa (2004) [35] | France | 101 | 101 | NS | NS | 0.4 | Serum | qPCR | SRY |
| Cremonesi (2004) [60] | Italy | 356 | 356 | 6 – 40 | 10 | 0.4 | Plasma | qPCR | SRY |
| Ho (2004) [83] | Singapore | 23 | 23 | 6 – 36 | 2 | 0.8 | Plasma | qPCR | SRY |
| Hwa (2004) [86] | Taiwan | 56 | 56 | 6 – 16 | 3 |  | Plasma | qPCR | SRY |
| Johnson (2004) [22] | USA | *63* | 315 | 10 – 20 | 20 | 0.8 | Plasma | qPCR | SRY |
| Ren (2004) [92] | China | 300 | 300 | 6 onwards | NS | 0.3 | Plasma | qPCR | SRY |
| Rijinders (2004) [74] | The Netherlands | 65 | 65 | 11 – 19 | 30 | 2 | Plasma | qPCR | SRY |
| Zhao (2004a) [100] | Japan | 44 | 44 | 9 – 22 | 20 | 0.2 | Plasma | Nested PCR | DYS14 |
| Zhao (2004b) [101] | China | 44 | 44 | 7 – 41 | 2 | 0.35 | Plasma | PCR | DYZ1 |
| Brojer (2005) [53] | Poland | 746 | 746 | 5 – 39 | 5 | 1 | Plasma | qPCR | SRY |
| Galbiati (2005) [8] | Italy | 1681 | 1681 | 6 – 40 | 10 | 0.4 | Plasma | qPCR | SRY |
| Hyett (2005) [67] | UK | 30 | 30 | 7 – 14 | 10 | 0.8 | Plasma | qPCR | SRY |
| Zhu (2005) [102] | China | 32 | 32 | 7 – 13 | 2-3 | 0.2 | Plasma | Nested PCR | Amelogenin |
| Zimmermann (2005) [27] | UK | 12 | 24 | 11 – 13 | 3.5 | 0.4 | Plasma | qPCR | SRY, DYS14 |
| Zolotukhina (2005) [103] | Russia | 60 | 60 | 9 – 28 | NS | 0.5 | Plasma and serum | Nested PCR | SRY |
| Chi (2006) [57] | UK | 10 | 11 | 11 – 14 | 15 | *0.8* | Plasma | qPCR | DYS14 |
| Davalieva (2006) [61] | Macedonia | 46 | 46 | 16 – 20 | 2-3 | 0.8 | Plasma | qPCR | SRY |
| Deng (2006) [24] | China | 64 | 64 | NS | 8-10 | 1.2 | Plasma | PCR | Y-STR (DYS) |
| Ge (2006) [82] | China | 76 | 76 | 4 – 16 | NS | 0.2 | Plasma | DNA microarray | SRY(1,2,3), DYS1, DYS14, DYZ3 |
| Hong (2006) [85] | China | 30 | 30 | NS | NS | 1.6 | Plasma | Nested PCR | SRY |
| Illanes (2006) [46] | UK | 20 | 20 | 11 – 14 | 20 | 0.8 | Plasma | qPCR | DYS14 |
| Jorgez (2006) [87] | USA | 15 | 86 | 10 – 29 | 20 | 0.8 | Plasma | qPCR | DYS1 |
| Li (2006) [47] | Switzerland | 97 | 97 | 7 – 40 | NS | 0.5 | Plasma | MALDI-TOF MS | SRY |
| Martinhago (2006) [33] | Brazil | 52 | 79 | 5 – 6 | 10 | 0.4 | Plasma | qPCR | DYS14 |
| Santacroce (2006) [94] | Italy | 40 | 40 | 7 – 30 | 9 | 0.5 | Plasma | qPCR | SRY, amelogenin |
| Stanghellini (2006) [77] | Italy | 57 | 107 | 5 – 12 | 9 | 0.3 | Serum | qPCR | DAZ4M8 |
| Zhong (2006) [81] | Switzerland | 12 | 12 | NS | 15 | 0.8 | Plasma | qPCR | SRY |
| Alberry (2007) [49] | Egypt and UK. | 15 | 15 | 8 – 13 | <10 | 1.1 | Plasma | qPCR | DYS14 |
| Boon (2007) [52] | UK | 58 | 44 | NS | NS | 0.8 | Plasma | qPCR | SRY |
| Chitty (2007) [7] | UK | 160 | 140 | NS | NS | NS | NS | qPCR | SRY OR DYS14 |
| Illanes (2007) [68] | UK | 26 | 26 | 11 – 15 | 5 | 0.8 | Plasma | qpCR | DYS14 |
| Lapaire (2007) [69] | Switzerland | 344 | 344 | 20 – 21 | NS | 0.8 | Plasma | qPCR | SRY, DYS14 |
| Liu (2007) [89] | China | 30 | 225 | 5 – 40 | 5 | 1 | Plasma | qPCR | SRY |
| Majer (2007) [71] | Austria | 151 | 151 | 32 – 40 | NS | 0.4 | Plasma | qPCR | SRY |
| Ren (2007) [93] | China | 150 | 150 | 5 – 9 | 2 | NS | Plasma | Nested PCR | SRY |
| Bustamante-Aragones (2008) [39] | Spain | 196 | 316 | 5 – 12 | 20 | 1 | Plasma | qPCR | SRY |
| Fan (2008) [13] | USA | 18 | 18 | 10 – 35 | 7-15 | 0.2 | Plasma | qPCR | DYS14 |
| Kimura (2008) [88] | Japan | 13 | 13 | 12 – 39 | 7 | 1 | Plasma | PCR | 17 Y-STR |
| Lazaros (2008) [20] | Greece | 37 | 37 | 7 – 9 | 5 | 5 | Serum | PCR | DYS14, DYZ1 |
| Minon (2008) [37] | Belgium | 545 | 545 | 10 – 38 | NS | 0.85 | Plasma | qPCR | SRY |
| Picchiassi (2008) [32] | Italy | 145 | 145 | 11 – 12 | 5 | 0.5 | Plasma | qPCR | DYS14, SRY |
| Tungwiwat (2008) [28] | Thailand | 168 | 168 | 5 – 32 | 2 | 0.2 | Plasma | Nested PCR | DYS14 |
| Urato (2008) [97] | USA | 113 | 113 | 15 – 21 | NS | 0.4 | Serum | qPCR | DYS1 |
| Vecchione (2008) [63] | Italy | 26 | 26 | NS | NS | 0.4 | Plasma | qPCR | Amelogenin |
| Vodicka (2008) [31] | Czech Republic | 475 | 475 | 4 – 37 | NS | NS | Plasma | PCR | Amelogenin |
| Wagner (2008) [98] | Croatia | 96 | 96 | 10 – 36 | NS | NS | Plasma | qPCR | SRY |
| Atamanuik (2009) [38] | Austria | 46 | 46 | NS | NS | 0.8 | Plasma | qPCR | SRY |
| Lapaire (2009) [40] | Germany | 406 | 406 | 20 – 21 | NS | 0.8 | Plasma | qPCR | SRY, DYS14 |
| Sesarini (2009) [41] | Argentina | 66 | 56 | 7 – 41 | 5 | 1 | Plasma | qPCR | DYS14 |
| Wang (2009) [44] | China | 78 | 78 | 14 – 40 | 10 | 0.2 | Plasma | qPCR | SRY |
| Sikora (2010) [42] | Switzerland | 51 | 51 | NS | NS | 0.5 | Plasma | qPCR | DYS14 |
| Vora (2010) [43] | USA | 52 | 52 | NS | NS | 0.4 | Plasma | qPCR | DYS1 |

NS – not stated

Supplementary Table 2: Summary statistics from posterior distributions from bivariate meta-regression analysis investigating the effect of week of test (standardized)

| **Covariates** | **Effect of covariates on sensitivity**  **Median (95% CI)** | **Effect of covariates on specificity**  **Median (95% CI)** |
| --- | --- | --- |
| Intercept | 3.34 (2.80, 4.04) | 3.83 (3.20, 4.66) |
| Week* | 0.41 (-0.13, 0.97) | 0.44 (-0.22, 1.09) |

* Covariate was standardized using mean 9.29 and standard deviation 2.34.

Supplementary Table 3: Summary statistics from posterior distributions from bivariate meta-regression analyses using all studies (n = number of 2×2 tables constructed from included studies).

|  | **Bivariate regressions with single covariate** | | | |  | **Bivariate regression with all covariates** | | | |
| --- | --- | --- | --- | --- | --- | --- | --- | --- | --- |
| **Covariates** | **Effect of covariates on sensitivity**  **Median (95% CI)** | | **Effect of covariates on specificity**  **Median (95% CI)** | |  | **Effect of covariates on sensitivity**  **Median (95% CI)** | | **Effect of covariates on specificity**  **Median (95% CI)** | |
| Plasma (n=75) | 3.20 | (2.84, 3.62) | 4.32 | (3.80, 4.96) |  |  |  |  |  |
| Serum (n=10) vs Plasma | 1.56 | (0.22, 3.03) | 1.82 | (-0.17, 4.34) |  | 1.38 | (0.01, 2.89) | 1.51 | (-0.31, 3.92) |
| SRY (n=53) | 3.41 | ( 2.94, 3.95) | 4.96 | (4.31, 5.73) |  |  |  |  |  |
| DYS 14 (n=14) vs SRY | -0.28 | (-1.21, 0.63) | -0.89 | (-2.08, 0.33) |  | 0.11 | (-0.84, 1.09) | 0.00 | (-1.16, 1.17) |
| other (n=19) vs. SRY | 0.08 | (-0.86, 1.01) | -1.47 | (-2.63, -0.34) |  | -0.04 | (-1.00, 0.94) | -1.25 | (-2.32, -0.22) |
| qPCR (n=58) | 3.57 | ( 3.11, 4.08) | 5.03 | (4.47, 5.69) |  |  |  |  |  |
| other (n=26) vs qPCR | -0.64 | (-1.37, 0.05) | -1.76 | (-2.57, -0.92) |  | -0.53 | (-1.36, 0.30) | -1.49 | (-2.44, -0.55) |
| Intercept (Volume) | 3.36 | (3.00, 3.79) | 4.61 | (4.08, 5.27) |  |  |  |  |  |
| Volume | 0.30 | (-0.14, 0.85) | 1.00 | (0.11, 2.15) |  | 0.21 | (-0.22, 0.74) | 0.54 | (-0.13, 1.46) |
| Intercept (Year) | 3.33 | (2.97, 3.75) | 4.47 | (3.95, 5.11) |  |  |  |  |  |
| Year | 0.36 | (0.01, 0.71) | 0.26 | (-0.28, 0.76) |  | 0.33 | (-0.03, 0.69) | 0.27 | (-0.18, 0.69) |

*The coefficients for these covariates relate to standardized covariate values.
